# Pitfalls in the diagnostics of shoulder dystocia: an analysis based on the scrutiny of 2274 deliveries

**DOI:** 10.1007/s00404-023-07022-8

**Published:** 2023-04-03

**Authors:** Karin Heinonen, Terhi Saisto, Mika Gissler, Marja Kaijomaa, Nanna Sarvilinna

**Affiliations:** 1 Department of Obstetrics and Gynecology, Helsinki University Hospital and University of Helsinki, Haartmaninkatu 2, 00290 Helsinki, Finland; 2 Department of Knowledge Brokers, THL Finnish Institute for Health and Welfare, Helsinki, Finland; 3 Region Stockholm, Academic Primary Health Care Centre, Stockholm, Sweden; 4 Department of Molecular Medicine and Surgery, Karolinska Institutet, Stockholm, Sweden

**Keywords:** Diagnostic descriptions, Incidence, Maneuvers, Guidelines, Erb’s palsy

## Abstract

**Purpose:**

Shoulder dystocia is an obstetric emergency with severe complications. Our objective was to evaluate the major pitfalls in the diagnostics of shoulder dystocia, diagnostic descriptions documented in medical records, use of obstetric maneuvers, and their correlations to Erb’s and Klumpke’s palsy and the use of ICD-10 code 066.0.

**Methods:**

A retrospective, register-based case–control study included all deliveries (*n* = 181 352) in Hospital District of Helsinki and Uusimaa (HUS) area in 2006–2015. Potential shoulder dystocia cases (*n* = 1708) were identified from the Finnish Medical Birth Register and the Hospital Discharge Register using ICD-10 codes O66.0, P13.4, P14.0, and P14.1. After thorough assessment of all medical records, 537 shoulder dystocia cases were confirmed. Control group consisted of 566 women without any of these ICD-10 codes.

**Results:**

The pitfalls in the diagnostic included suboptimal following of guidelines for making the diagnosis of shoulder dystocia, subjective interpretation of diagnostic criteria, and inexact or inadequate documentation in medical records. The diagnostic descriptions in medical record were highly inconsistent. The use of obstetric maneuvers was suboptimal among shoulder dystocia cases (57.5%). Overall, the use of obstetric maneuvers increased during the study period (from 25.7 to 97.0%, *p* < 0.001), which was associated with decreasing rate of Erb’s palsy and increasing use of ICD-10 code O66.0.

**Conclusion:**

There are diagnostic pitfalls, which could be addressed by education regarding shoulder dystocia guidelines, by improved use obstetric maneuvers, and more precise documentation. The increased use of obstetric maneuvers was associated with lower rates of Erb’s palsy and improved coding of shoulder dystocia.

## What does this study add to the clinical work

**Table:** 

The major pitfalls in the diagnostics of shoulder dystocia included suboptimal following of shoulder dystocia guidelines, subjective interpretation of diagnostic criteria, and inexact or inadequate documentation in medical records. These challenges could be tackled with education on guidelines, improved use of obstetric maneuvers, and more precise documentation.	

## Introduction

Shoulder dystocia is a frightening complication of vaginal delivery. It has been associated with major maternal and neonatal morbidities [1, 2]. The guidelines of the Royal College of Obstetricians and Gynaecologists (RCOG) and the American College of Obstetricians and Gynecologists (ACOG) define shoulder dystocia as vaginal vertex delivery complicated by the inability to deliver the shoulders with gentle downward traction, and requiring additional obstetric maneuvers [3, 4]. The incidence ranges between 0.1 and 3.0% [2, 5]. Much of the variation in the reported incidence rates is likely due to subjective clinical judgment and the varying diagnostic descriptions of shoulder dystocia [6]. Suggested definitions of shoulder dystocia include tight shoulders, difficulty in delivering the shoulders, clinical judgment, head-to-body delivery time ≥ 60 s, failure of delivering shoulders with gentle downward traction, and delivery requiring additional obstetric maneuvers [6, 7].

The objective of this study was to identify some of the problems in the diagnostics of shoulder dystocia. We suspected shoulder dystocia to be underrecoded and wanted to evaluate how accurately ICD-10 code O66.0 (obstructed labor due to shoulder dystocia) is being used. In the published literature, the incidence of shoulder dystocia is usually reported based on the diagnosis code in medical records, but it has been rarely specified, which diagnostic descriptions have been used [8–22]. We hypothesized that diagnostic descriptions could be highly variable and wanted to assess, which descriptions were found in the shoulder dystocia cases of our study population and how they correlated with international guidelines.

We suspected that shoulder dystocia’s ICD-10 code would not necessarily cover all shoulder dystocia cases, and therefore, we also scrutinized all medical records with possible shoulder dystocia-related diagnoses (clavicle fracture, Erb’s palsy, and Klumpke’s palsy) to uncover potential underdiagnostics. We also wanted to evaluate the use of obstetric maneuvers and how it correlated with the rates of Erb’s palsy and ICD-10 code O66.0.

## Materials and methods

**Fig. 1:**
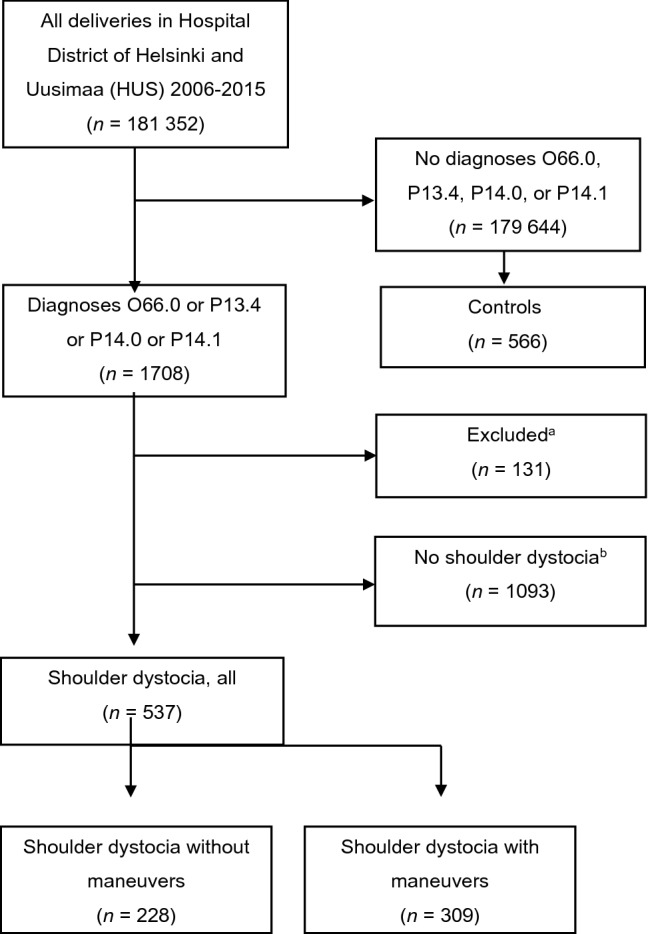
Flowchart of the research data. O66.0, Shoulder dystocia; P13.4, fracture of clavicle due to birth injury, P14.0, Erb’s palsy due to birth injury; P14.1, Klumpke’s palsy due to birth injury.^a^P13.4 or/and P14.0 diagnosis found in cesarean sections, stillbirths, or breech deliveries, and cases with deficient or incorrect data.^b^Medical records were read thoroughly, and no features of shoulder dystocia were found

Our register-based study included all deliveries in Hospital District of Helsinki and Uusimaa (HUS) between 2006 and 2015 (*n* = 181 352, Fig. 1). Potential shoulder dystocia cases were found using data from the Finnish Medical Birth Register and the Hospital Discharge Register. Both registers are maintained by the Finnish Institute for Health and Welfare and all live births and stillbirths with gestational age of ≥ 22 weeks or birth weight of ≥ 500 g are included, as well as all hospital inpatient and outpatient episodes. Good quality of these Finnish registers has been shown in earlier studies [23, 24].

We searched for ICD-10 codes O66.0 (obstructed labor due to shoulder dystocia), P13.4 (fracture of clavicle due to birth injury), P14.0 (Erb’s palsy due to birth injury), and P14.1 (Klumpke’s palsy due to birth injury) to identify potential shoulder dystocia parturients and their children. Children’s diagnoses were searched until 1 year of age to identify cases with permanent brachial plexus injury. In Finland, the ICD-10 classification was introduced in 1996 and these diagnosis codes are added to medical records by obstetrician, midwife, or pediatrician. Controls were also obtained from the Medical Birth Register, but without any of those abovementioned ICD-10 codes. Controls matched by mode of delivery, gestational age, maternal age, year of index birth, and parity were randomly selected among all deliveries during study period. Only singleton pregnancies were included.

All medical records of potential shoulder dystocia cases were reviewed thoroughly by clinically experienced obstetricians (K.H., T.S., N.S.). We also scrutinized medical records of the controls to make sure that no misclassification for shoulder dystocia was included in that group. Features, which we considered as strongly supportive of the correct diagnosis, included the definitions of shoulder dystocia listed above in Introduction, the ICD-10 code O66.0, suspected Erb’s palsy immediately after difficult or tight delivery of shoulders, and the Turtle sign.

We analyzed maternal, labor-related, and neonatal factors in all shoulder dystocia cases and compared them to controls. Separate analyses were also made for nulliparous and multiparous parturients. We also compared shoulder dystocia cases with or without obstetric maneuvers and cases with or without registered the ICD-10 code O66.0 to look for possible differences and therefore possible risk factors for undercoding and for not using required maneuvers between these subgroups. Cases without unambiguously named maneuvers or otherwise deficiently documented situations were classified into the without maneuvers group. In the shoulder dystocia group, the descriptions of shoulder dystocia found in medical records were evaluated following the definitions presented in Introduction [6, 7].

### Statistical analyses

The Chi‐square test and Fisher’s Exact test were used to test the statistical significance of categorical variables. For continuous variables, either Mann–Whitney *U* test or Independent Samples *t* test was used. The test for relative proportions for two samples was used to test the statistical significance of the changes in the incidences during the study period. ORs with 95% CI were calculated for binary outcomes using the control group, shoulder dystocia cases without maneuvers, or shoulder dystocia cases without the ICD-10 code O66.0 as a reference. The statistical analyses were performed using IBM SPSS Statistics (version 28).

## Results

There were 1708 identified cases of potential shoulder dystocia. After detailed review, 1093 cases with either isolated clavicle fracture or Erb’s palsy without concurrent shoulder dystocia were found; therefore, they were excluded from the data (Fig. 1). Of those, 131 cases were excluded because the medical record data was either deficient or clavicle fracture was diagnosed after cesarean section or stillbirth (Fig. 1). Shoulder dystocia was not diagnosed in newborns born by cesarean sections and Erb’s palsy was diagnosed after cesarean section in only one case. Features, which were strongly supportive of the correct diagnosis of shoulder dystocia were found in 537 cases. We divided these 537 cases into two subgroups as presented in Fig. 1: shoulder dystocia without maneuvers (*n* = 228) and shoulder dystocia with maneuvers (*n* = 309).

Table 1 includes the descriptions of shoulder dystocia found in the medical records using terminology presented in the Introduction [6, 7]. The most often mentioned description was “tight shoulders” (96.8%). The use of additional obstetric maneuvers was found in 57.5% of the cases. Altogether 48.9% (151/309) received the ICD-10 code O66.0 when obstetric maneuvers were used. A trend to use only term “tight shoulder” instead of diagnosing shoulder dystocia was observed in group without maneuvers. The reported obstetric maneuvers are listed in Table 2. McRoberts’ maneuver was used most frequently (in 88.0%). We found medical records with poor documentation where unnamed or otherwise unexplained obstetric maneuvers were used. Cases with these deficiencies (*n* = 228) were classified as in the group without maneuvers.

**Table 1:** Observed descriptions of shoulder dystocia in medical records. Data are percentages (%)

Description	Shoulder dystocia, all (*n* = 537)	Shoulder dystocia with maneuvers (*n* = 309)	Shoulder dystocia without maneuvers (*n* = 228)
Tight shoulders	96.8	98.1	95.2
Additional obstetric maneuvers^a,b^	57.5	100	0
Difficulty to deliver shoulders after the head is delivered	54.0	70.6	31.6
Suspected Erb’s palsy immediately after difficult/tight delivery of shoulders	46.2	44.0	49.1
Head-to-body delivery time			
Reported	11.4	18.1	2.2
Over 60 s	9.9	15.6	2.2
Turtle sign	8.0	11.7	3.1
Downward traction fails to deliver shoulders^a,b^	6.7	11.0	0.9
Diagnosis code O66.0 used	31.7	48.9	8.3

*NB* Multiple descriptions could be observed in a single shoulder dystocia case, *RCOG* the Royal College of Obstetricians and Gynaecologists, *ACOG* the American College of Obstetricians and Gynecologists

^a^Included to RCOG definition of shoulder dystocia

^b^Included to ACOG definition of shoulder dystocia

**Table 2:** Shoulder dystocia maneuvers mentioned in medical records. Data are number of cases and percentages *(n* %)

Shoulder dystocia maneuver	Shoulder dystocia with maneuvers (*n* = 309)
McRoberts’ maneuver	272 (88.0)
Suprapubic pressure	146 (47.2)
Internal rotational maneuvers^a^	69 (22.3)
Delivering of the posterior arm	34 (11.0)
All fours	3 (1.0)
Symphysiotomy	1 (0.3)
Attempted abdominal rescue^b^	1 (0.3)

^a^Includes Woods corkscrew maneuver and Rubin maneuver

^b^The shoulders were delivered before the incision to the uterus was made under general anesthesia in operating room

**Fig. 2:**
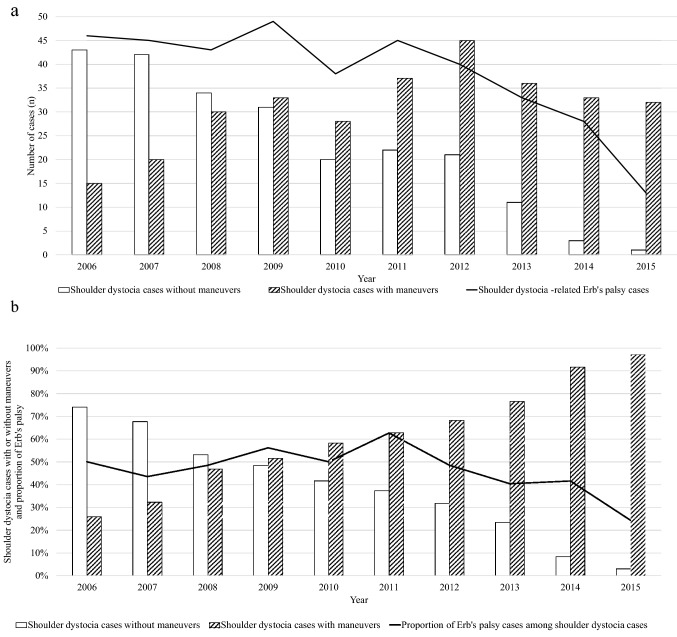
**a** The numbers of shoulder dystocia cases (with or without obstetric maneuvers) and shoulder dystocia-related Erb’s palsy cases in total in Hospital District of Helsinki and Uusimaa (HUS) in 2006–2015. **b** The use of obstetric maneuvers and proportion of Erb’s palsy among shoulder dystocia cases in Hospital District of Helsinki and Uusimaa (HUS) in 2006–2015

Figure 2a, b presents the annual changes in the numbers and proportions of shoulder dystocia cases (with and without obstetric maneuvers) and shoulder dystocia-related Erb’s palsy. Figure 2b shows significant increase in the use of obstetric maneuvers in shoulder dystocia cases during the study period (from 25.7 to 97.0%, *p* < 0.001). Concurrently, proportion of Erb’s palsy cases among shoulder dystocia declined from 50.0 to 24.2% (*p* = 0.008) (Fig. 2b). During the study period, the incidence of ICD-10 code O66.0 for shoulder dystocia increased from 0.04 to 0.15% (*p* < 0.001) and the incidence of shoulder dystocia-related Erb’s palsy decreased from 0.16 to 0.05% (*p* < 0.001). In addition, the proportion of shoulder dystocia cases having received the ICD-10 O66.0 rose from 12.1 to 81.8% (*p* < 0.001). During the study period, the rate of cesarean deliveries in Finland varied between 15.9 and 16.7%, with no statistically significant trend between 2006 and 2015 (16.3 vs 15.9%, *p* = 0.067).

The overall incidence of the ICD-10 code O66.0 in this study population was 0.09% (170/181 352). After thorough scrutinization of the medical records including ICD-10 codes related to shoulder dystocia (P13.4, P14.0 and P14.1), the incidence of shoulder dystocia increased over threefold to 0.30% (537/181 352). Altogether only 31.7% (170/537) of these confirmed shoulder dystocia cases had received the ICD-10 code O66.0.

There were 566 controls selected from the Medical Birth Register list. Matching was successful as there were no statistically significant differences between groups regarding vacuum extractions (29.4 vs. 28.4%, *p* = 0.32), maternal age (mean 30.2 vs. 30.3, *p* = 0.80), gestational age (mean 282 d vs. 282 d, *p* = 0.24), or the proportion of late and post-term pregnancies (gestational age ≥ 41 + 0 weeks) (36.7 vs. 35.2%, OR 1.07, 95% CI 0.84–1.37). There were no forceps deliveries in this dataset. Maternal and labor-related characteristics, and neonatal outcomes of shoulder dystocia cases and controls are presented in Table 3. There were several substantial differences between shoulder dystocia cases and controls: cases had more likely BMI ≥ 30 (OR 1.66, 95% CI 1.14–2.42), gestational diabetes (GDM) (OR 2.09, 95% CI 1.45–3.01), or any type of diabetes (OR 2.72, 95% CI 1.92–3.87) (Table 3). Shoulder dystocia deliveries were more often induced (OR 1.66, 95% CI 1.27–2.18) (Table 3). This difference remained, when nulliparous and multiparous women were analyzed separately (Table 3). Among the shoulder dystocia cases, 48.0% (258/537) had Erb’s palsy and 46.4% (249/537) had a clavicular fracture (Table 3). There were no cases of Klumpke’s palsy. As shown in Table 3, shoulder dystocia infants were considerably larger in birthweight on average (4190 g vs. 3582 g,* p* < 0.001).

**Table 3:** Maternal and labor-related characteristics, and neonatal outcomes of shoulder dystocia cases and controls. Data are percentages (%) unless otherwise specified

Variable	Shoulder dystocia (*n* = 537)	Controls (*n* = 566)	OR/mean difference^a^ (95% CI)	*p*
Nulliparous	44.7	39.2	1.25 (0.99–1.59)	0.067
Multiparous	55.3	60.8	0.80 (0.63–1.02)	0.067
BMI ≥ 30	15.0	9.6	1.66 (1.14–2.42)	0.009
Gestational diabetes	17.1	9.0	2.09 (1.45–3.01)	< 0.001
Any diabetes type	21.6	9.2	2.72 (1.91–3.87)	< 0.001
Induction of labor	30.9	21.2	1.66 (1.27–2.18)	< 0.001
–Nulliparous	31.7	20.3	1.82 (1.19–2.79)	0.006
–Multiparous	30.3	21.8	1.56 (1.09–2.23)	0.015
Erb’s palsy	48.0	0.0	NA	NA
Clavicle fracture	46.4	0.0	NA	NA
Birthweight (g) *mean* ± *SD*,* (range)*	4190 ± 431 (2692–5778)	3582 ± 452 (2280–4900)	609 (557–661)^a^	< 0.001

*NA* not applicable

All *p* values were calculated by comparing shoulder dystocia cases to controls

^a^Mean difference

There were a few statistically significant differences between shoulder dystocia cases with and without maneuvers (Table 4): any type of diabetes was more frequent in shoulder dystocia cases with maneuvers (OR 1.69, 95% CI 1.10–2.60) and they had more vacuum extractions (OR 1.89, 95% CI 1.32–2.70). The incidence of clavicle fractures (34.6% vs. 62.3%, OR 0.32, 95% CI 0.22–0.45) was significantly lower with maneuvers (Table 4). The same but statistically insignificant trend was seen with the incidence of Erb’s palsy (45.0% vs. 52.2%, OR 0.75, 95% CI 0.53–1.05). There were no statistically significant differences in birthweight between shoulder dystocia cases with or without maneuvers (Table 4).

**Table 4:** Maternal and labor-related characteristics and neonatal outcomes of shoulder dystocia cases with and without maneuvers. Data are percentages (%) unless otherwise specified

Variable	Shoulder dystocia with maneuvers *n* = 309)	Shoulder dystocia without maneuvers (*n* = 228)	OR/mean difference^a^ (95% CI)	*p*
Nulliparous	47.2	41.2	1.28 (0.90–1.80)	0.19
Multiparous	52.8	58.2	0.78 (0.55–1.11)	0.19
Any diabetes type	25.2	16.7	1.69 (1.10–2.60)	0.019
Vacuum extraction	47.6	32.5	1.89 (1.32–2.70)	0.001
–Nulliparous	72.6	57.4	1.96 (1.14–3.39)	0.017
–Multiparous	25.2	14.9	1.91 (1.06–3.46)	0.031
Erb’s palsy	45.0	52.2	0.75 (0.53–1.05)	0.097
Clavicle fracture	34.6	62.3	0.32 (0.22–0.45)	< 0.001

NA, not applicable

All *p* values were calculated by comparing shoulder dystocia cases with maneuvers to shoulder dystocia cases without maneuvers

^a^Mean difference

The shoulder dystocia cases with and without the ICD-code O66.0 were also compared with each other. When ICD-10 code O66.0 was given, vacuum extraction had been performed more often in both nulliparous (76.9% vs. 61.7%, OR 2.07, 95% CI 1.12–3.82) and multiparous women (31.5% vs. 15.6%, OR 2.49, 95% CI 1.39–4.44). Notably, the obstetric maneuvers were used more often with the ICD-10 code O66.0 (88.8% vs. 43.1%, OR 10.51, 95% CI 6.25–17.68) and the average number of maneuvers was higher (2.1 vs. 0.7, *p* < 0.001). Otherwise, there were no differences between these two groups.

## Discussion

Our study showed that when the shoulder dystocia guidelines are not followed adequately, there is a tendency to be subjective, not to use clear diagnostic criteria, and to provide imprecise documentation in medical records, all of which were major pitfalls in the diagnostics of shoulder dystocia. The increased use of appropriate obstetrical maneuvers was associated with a decrease in the incidence of Erb’s palsy and clavicle fractures and an increase in the appropriate use of ICD-10 code O66.0. One of the challenges was the use of term “tight shoulders” instead of formally diagnosing shoulder dystocia even when obstetric maneuvers were used. This reflects the insufficient knowledge and/or use of RCOG and ACOG guidelines. Moreover, the use of obstetric maneuvers altogether was suboptimal among our shoulder dystocia cases (57.5%), perhaps reflecting high threshold for using maneuvers. Fortunately, as shown in Fig. 2a, b, significant improvement was noted in the use of obstetric maneuvers and a positive change in practice towards the recommendations of RCOG and ACOG during our study period.

Our study demonstrated major undercoding of shoulder dystocia even if obstetric maneuvers had been used. Out of 537 shoulder dystocia cases, 68.3% did not have the ICD-10 code O66.0. There was a threefold difference between the incidence of the ICD-10 code O66.0 (0.09%) and the incidence of shoulder dystocia based on scrutiny of medical records (0.30%). Our results suggest that the incidences of shoulder dystocia found in the published literature should be interpreted with extreme caution since substantial undercoding may be involved. Undercoding is a known phenomenon with diagnostic coding, but our results help to estimate the extent of undercoding related to shoulder dystocia. Despite significant undercoding in this dataset, the incidence of shoulder dystocia in our hospitals was overall low (0.30%), which is similar to the overall incidence in Finland in 2017 (0.32%) [25] and comparable with other Nordic studies (0.11–0.73%) [8, 26]. We could not identify any subgroup prone to undercoding or underdiagnostics. Thus, receiving the appropriate ICD-10 code O66.0 seems to depend on the clinician’s subjective assessment. However, vacuum extraction and maternal diabetes were correlated with an increased use of obstetric maneuvers. This in turn was associated with higher rates of ICD-10 code O66.0. Perhaps the threshold for using obstetric maneuvers was intuitively lower when these risk factors for shoulder dystocia were present.

Our results give a novel insight into diagnostic challenges of shoulder dystocia, which have not been studied in this type of setting before even though the impact of methodology on the incidence rates has been recognized and acknowledged [5]. Our way of collecting information by reading through medical records was highly more detailed compared to data collection from registers only, but it was too laborious to be used in clinical practice or in larger data sets. Many studies have relied on diagnosis codes found in medical records [8–15], while two studies had a specific field in their databases for reporting, if delivery was complicated by shoulder dystocia [26, 27]. Several previous studies did not describe the criteria for selection of shoulder dystocia cases [16–21, 28]. The majority did not mention, which definition(s) were used, while some described that it depended on the judgment of the clinician in charge [8–22]. RCOG’s definition of shoulder dystocia or requirement of obstetric maneuvers was used in two previous studies only [27, 28].

Both ACOG and RCOG guidelines define shoulder dystocia as vaginal delivery, which requires additional obstetric maneuvers to deliver shoulders after a failed downward traction [3, 4], which is a considerably narrower definition than all diagnostic descriptions listed in Introduction. Our shoulder dystocia cases included a wide range of descriptions, which were not always in line with the RCOG and ACOG recommendations. Tight shoulders were mentioned almost every time even though it is not essential for diagnosis according to ACOG and RCOG guidelines [3, 4]. A recent Finnish study reported that implementing regular simulation trainings decreased the use of term “tight shoulders” instead of shoulder dystocia diagnosis [29], and this trend was also observed in our dataset. Turtle sign was reported only in 8.0%, which supports its role as a suggestive sign of shoulder dystocia but not a diagnostic one [4]. Objective head-to-body delivery time turned out to be problematic, because it was infrequently recorded and not routinely measured. It has also been linked to risk of overdiagnosis [30], which in turn could lead to an unnecessary increase in cesarean section rates in subsequent pregnancies.

There was an obvious association between increased use of obstetric maneuvers and decreased Erb’s palsy rates (Fig. 2), which has also been reported in earlier studies [27, 29]. In addition, there were statistically significantly less clavicle fractures when obstetric maneuvers were used. Therefore, obstetricians and midwifes should not hesitate to use obstetric maneuvers, if shoulder dystocia is present or suspected. Particularly the total absence of obstetric maneuvers endangers the infant for complications and results in undercoding, which was also and unfortunately observed in some of our cases. After all, the correct diagnosis has a major effect on the follow-up und treatment of future pregnancies and labors. In addition to using obstetric maneuvers, the precise recording of them in medical records is crucial. Fortunately, a positive trend towards more frequent use of obstetric maneuvers and improved documentation was observed in our data. We suggest that all medical record systems should, at minimum, require the registration of shoulder dystocia status (yes/no) and the used maneuvers (or their absence) in every labor and delivery. This would improve not only the accuracy of diagnostics, but also has the potential to decrease complications of shoulder dystocia.

### Strengths and limitations

In this study, we gathered 10 years’ worth of data containing all births in the largest hospital district of Finland and we accessed the medical records; therefore, we were able to get more precise information on the study population than would be possible using registers only. Medical records of 2274 parturients and their children were reviewed in their entirety. These detailed data have enabled a scrutiny of the shoulder dystocia descriptions used and, therefore, provided a unique insight into hazards of the diagnostic process that, to our knowledge, has not been reported in earlier studies. Our study suggests that undercoding influences the accuracy of published incidence rates as those are based on diagnosis coding. The fact that we were able to divide the shoulder dystocia cases into subgroups allowed us to demonstrate the variability that is inherent to making this diagnosis.

Since we used ICD-10 codes to search for shoulder dystocia cases, selection bias and coding errors could have affected our results. Due to the method of selection of cases and controls, we probably did not recognize all unrecorded shoulder dystocia cases, especially those without complications. Because of our complication-based search, the rates of complication among shoulder dystocia cases are emphasized, even though the overall rates of Erb’s palsy (total 0.21%, shoulder dystocia-related 0.14%) and clavicle fracture (total 0.67%, shoulder dystocia-related 0.14%) were low in our study population.

## Conclusions

The major pitfalls in diagnostics of shoulder dystocia include inexact documentation, subjective interpretation of diagnostic criteria, and not following the recommended guidelines. The increased use of obstetric maneuvers was associated with lower rates of Erb’s palsy and better recognition of shoulder dystocia. Education, including better knowledge of ACOG and RCOG guidelines, could increase the use of appropriate obstetric maneuvers and, therefore, lead to improved neonatal outcomes as well as to more precise documentation in medical records. Electronic medical record programs/software should contain sections, which would automatically require and help the end user to provide the correct documentation.

## Data Availability

The data from the Finnish registers and medical records have been collected for this specific study, and this data cannot be distributed without authorization from the registers keepers.
